# Home-based versus outpatient pulmonary rehabilitation program for patients with chronic obstructive pulmonary disease: A protocol for systematic review and meta-analysis

**DOI:** 10.1097/MD.0000000000026099

**Published:** 2021-05-28

**Authors:** Guiyun Shi, Chuanjun Chen

**Affiliations:** a Department of Geriatric; b Department of Oncology, Xinchang County People's Hospital, Zhejiang 312500, China.

**Keywords:** chronic obstructive pulmonary disease, home-based pulmonary rehabilitation, meta-analysis, protocol, systematic review

## Abstract

**Background:**

Although home-based pulmonary rehabilitation programs have been shown in some studies to be an alternative and effective model, there is a lack of consensus in the medical literature due to different study designs and lack of standardization among procedures. Therefore, the purpose of this study was to compare the efficacy of a home-based versus outpatient pulmonary rehabilitation program for patients with chronic obstructive pulmonary disease (COPD).

**Methods:**

Five electronic databases including Embase, PubMed, Scopus, Science Direct, and Cochrane Library will be searched in May 2021 by 2 independent reviewers. The reference lists of the included studies will be also checked for additional studies that are not identified with the database search. There is no restriction on the dates of publication or language in the search. The randomized controlled trials focusing on comparing home-based and outpatient pulmonary rehabilitation for COPD patients will be included in our meta-analysis. The following outcomes should have been measured: functional exercise capacity, disease-specific health-related quality of life, and cost-effectiveness measures. Risk ratio with a 95% confidence interval or standardized mean difference with 95% CI is assessed for dichotomous outcomes or continuous outcomes, respectively.

**Results:**

It was hypothesized that these 2 methods would provide similar therapeutic benefits.

**Registration number:**

10.17605/OSF.IO/5CV48.

## 1 Introduction

Chronic obstructive pulmonary disease (COPD) is characterized by restricted airflow, which leads to a decreased ability to ventilate, and is associated with shortness of breath. Patients with severe airflow restriction and those who experience repeated acute exacerbations often suffer from reduced quality of life, impaired exercise ability, and an increased risk of readmission to hospital.^[1]^

Pulmonary rehabilitation is a multidisciplinary intervention that combines nutritional treatment, psychological support, physical exercise, and patient education.^[2]^ Intervention designed to accelerate post-hospitalization recovery and improve symptoms may lead to reduced use of healthcare in the future and to real improvements in quality of life and functional capacity in patients with shortness of breath and vulnerable COPD. However, the use of pulmonary rehabilitation therapy is generally low although strong scientific advice for its routine use in the treatment of COPD.^[3,4]^

Home-based unsupervised pulmonary rehabilitation therapy has been proposed as an alternative model that can increase access and absorption while curbing the rising health care costs associated with COPD. Although home-based pulmonary rehabilitation programs have been shown in some studies to be an alternative and effective model, there is a lack of consensus in the medical literature due to different study designs and lack of standardization among procedures.^[5,6]^ In addition, since most studies have been conducted under remote supervision, such as home visits or phone calls from physiotherapists or doctors, there is limited data on unsupervised rehabilitation programs.^[7–9]^ Therefore, the purpose of this study was to compare the efficacy of home-based versus outpatient pulmonary rehabilitation programs for patients with COPD. It was hypothesized that these 2 methods would provide similar therapeutic benefits.

## 2 Materials and methods

### 2.1 Protocol registration

The prospective registration has been approved by the Open Science Framework (OSF) registries (https://osf.io/5cv48), and the registration number is 10.17605/OSF.IO/5CV48. The protocol was written following the Preferred Reporting Items for Systematic Reviews and Meta-Analyses Protocols statement guidelines.

### 2.2 Selection of studies

Five electronic databases including Embase, PubMed, Scopus, Science Direct, and Cochrane Library will be searched in May 2021 by 2 independent reviewers. The reference lists of the included studies will be also checked for additional studies that are not identified with the database search. There is no restriction on the dates of publication or language in the search. No ethical approval is required in our study because all analyses are based on aggregate data from previously published studies.

### 2.3 Inclusion and exclusion criteria

The randomized controlled trials focusing on comparing home-based and outpatient pulmonary rehabilitation for COPD patients will be included in our meta-analysis. The following outcomes should have been measured: functional exercise capacity, disease-specific health-related quality of life, and cost-effectiveness measures. The exclusion criteria contain biochemical trials, reviews, case reports, no assessment of outcomes mentioned above, and no comparison of home-based and outpatient pulmonary rehabilitation for COPD patients.

### 2.4 Data extraction

A standard data extraction form is used independently by 2 reviewers to retrieve the relevant data from the articles. These variables include author, study design, sample size, publishing date, population, type of interventions and controls, follow-up, and outcomes. The outcome measures are as following: functional exercise capacity, disease-specific health-related quality of life, and cost-effectiveness measures. Data extraction is performed independently, and any conflict is resolved before final analysis. If data are not presented in the original article, corresponding authors will be contacted to acquire the missing data.

### 2.5 Quality assessment

The Grading of Recommendations Assessment, Development and Evaluation system will be used by 2 independent reviewers to rate the overall quality of evidence in each pooled analysis. The following 7 items will be used to assess the quality of randomized controlled trials: random sequence generation, allocation concealment, blinding of participants and personnel, blinding of outcome assessment, incomplete outcome data, selective reporting, and other bias. The quality rating high is reserved for evidence based on randomized controlled trials. The quality rating moderate, low, or very low are rated depending on the following 4 factors: risk of bias, inconsistency of effect, imprecision, and indirectness. When the heterogeneity is high, inconsistency will be considered serious. When there are fewer than 400 participants for each outcome, imprecision will be considered an appreciable risk. Any controversy will be resolved by discussing with a third author to reach a final consensus.

### 2.6 Statistical analysis

Review Manager software (v 5.4; Cochrane Collaboration) is used for the meta-analysis. Extracted data are entered into Review Manager by the first independent author and checked by the second independent author. Risk ratio with a 95% confidence interval or standardized mean difference with 95% CI is assessed for dichotomous outcomes or continuous outcomes, respectively. The heterogeneity is assessed using the *Q* test and *I*^2^ statistic. An *I*^2^ value of <25% is chosen to represent low heterogeneity and an *I*^*2*^ value of >75% to indicate high heterogeneity. All outcomes are pooled on random-effect model. A *P* value of <0.05 is considered to be statistically significant.

## 3 Discussion

Home-based unsupervised pulmonary rehabilitation therapy has been proposed as an alternative model that can increase access and absorption while curbing the rising health care costs associated with COPD. Although home-based pulmonary rehabilitation programs have been shown in some studies to be an alternative and effective model, there is a lack of consensus in the medical literature due to different study designs and lack of standardization among procedures.^[5,6]^ In addition, since most studies have been conducted under remote supervision, such as home visits or phone calls from physiotherapists or doctors, there is limited data on unsupervised rehabilitation programs.^[7–9]^ Therefore, the purpose of this study was to compare the efficacy of home-based versus outpatient pulmonary rehabilitation programs for patients with COPD. It was hypothesized that these two methods would provide similar therapeutic benefits.

## Author contributions

**Conceptualization:** Guiyun Shi.

**Data curation:** Guiyun Shi, Chuanjun Chen.

**Formal analysis:** Guiyun Shi, Chuanjun Chen.

**Investigation:** Guiyun Shi, Chuanjun Chen.

**Methodology:** Chuanjun Chen, Guiyun Shi.

**Resources:** Chuanjun Chen.

**Software:** Guiyun Shi.

**Supervision:** Chuanjun Chen, Guiyun Shi.

**Validation:** Chuanjun Chen.

**Writing – original draft:** Guiyun Shi.

**Writing – review & editing:** Chuanjun Chen.
